# Effects of Different Papua New Guinea Sweetpotato Varieties on Performance and Level of Enteric Pathogens in Chickens

**DOI:** 10.3390/ani9040188

**Published:** 2019-04-23

**Authors:** Janet Pandi, Phil Glatz, Rebecca Forder, Kapil Chousalkar

**Affiliations:** 1 School of Animal and Veterinary Sciences, Roseworthy Campus, University of Adelaide, Roseworthy SA 5371, Australia; janet.pandi@nari.org.pg (J.P.); bec.forder@adelaide.edu.au (R.F.); 2 South Australian Research and Development Institute, Roseworthy Campus, University of Adelaide, Roseworthy SA 5371, Australia; pntglatz@gotalk.net.au

**Keywords:** Poultry, *Salmonella* spp., *Campylobacter*, *Clostridium* spp., sweetpotato

## Abstract

**Simple Summary:**

The smallholder poultry industry in Papua New Guinea (PNG) has grown rapidly in the last decade. The cost of growing meat birds is high, as feed alone makes up to 80% of the total cost of production in PNG. Sweetpotato is currently used in a poultry feed as a cheaper alternative option compared to the more expensive commercially manufactured stockfeed in PNG. The PNG smallholder poultry production system involves many families who rear multiple batches of meat birds every year. These birds are sold mostly at the farm gate, at local provincial markets, or roadside markets. Consumption of contaminated chicken meat has been identified as one of the important food vehicles for food borne illness. This PNG-based study was conducted to understand whether the inclusion of local sweetpotato in poultry feed can influence the shedding of pathogens such as *Clostridium perfringens*, *Salmonella* and *Campylobacter* without causing negative effects on poultry performance. The results of this study releveled that *Campylobacter* and *Salmonella* levels in the broilers fed with the local sweetpotato diets can be influenced with inclusion of enzymes in the feed.

**Abstract:**

In the last decade, research has targeted the evaluation of local feed ingredients for use in monogastric diets to alleviate the high cost of production of livestock at smallholder levels in Papua New Guinea (PNG). The PNG smallholder poultry production system involves many families who rear multiple batches of meat birds every year. This study was conducted to evaluate the levels of enteric pathogens in the caeca of broilers fed with sweetpotato diets with varying levels of non-starch polysaccharides (NSP). Selection of a sweetpotato variety for use in broiler diets should be based on the total NSP content. In particular, varieties with low soluble NSPs are economical to use as Apparent Metabolizable Energy (AME) values are within the desired range for poultry and there is minimal need to include enzymes to improve NSP digestibility. The use of varieties with a low total NSP is also advantageous as the numbers of *Clostridium perfringens* was lower in broilers fed with these sweetpotato varieties. The level of *Campylobacter* and *Salmonella* levels were high in the ceca of birds fed with the sweetpotato varieties with high total NSP. These levels can be reduced with the inclusion of enzymes. This information will assist in the efficient use of local varieties of sweetpotato in PNG by small holder poultry farmers for sustainable poultry production and the commercial industry.

## 1. Introduction

The smallholder broiler industry in Papua New Guinea (PNG) has grown rapidly in the last decade and in 2013 was estimated to be worth around AUS$104 million/annum. In addition, the smallholder broiler sector is earning about 39.3% more than the commercial frozen carcass industry in PNG. The cost of growing poultry is high, as feed alone makes up to 80% of the total cost of production in PNG. Sweetpotato is currently used in a finisher ration for broiler chickens as a cheaper alternative option to the more expensive manufactured finisher stockfeed in PNG. Use of non-conventional ingredients in diets for monogastric livestock is limited due to high levels of dietary fiber [1]. Dietary fiber is the sum of non-starch polysaccharides (NSPs) and lignin and is a significant part of plant material [2]. The NSPs are either soluble or insoluble depending on their chemical properties. The soluble component has been known to exhibit anti-nutritive activities in pigs and poultry, leading to negative changes in gut physiology, microflora, and gut health, while the insoluble proportion imparts beneficial effects on gut development and secretion of endogenous digestive enzymes [2]. Sweetpotato has been used in the diets of fish, pigs and poultry to substitute grains, due to its availability and high carbohydrate (starch) content [3]. Sweetpotato has been promoted as a cheaper alternative energy source for poultry, especially for broilers destined for the live broiler chicken markets in PNG [3]. The PNG smallholder broiler production system involves more than 50,000 families who produce up to 6 batches of broilers yearly, growing birds up to 42 days of age. These birds are sold mostly at the farm gate, at local provincial markets, or roadside markets. This enterprise is mainly family owned at village level and the owners understanding of the implementation of biosecurity and food safety is highly variable. Chickens can harbor foodborne pathogens such as *Salmonella* and *Campylobacter*. Chickens closely interact with humans in the same household and thus potentially expose family members of smallholder poultry farmers to enteric pathogens due to their close contact with poultry. There has been no study which directly links chicken meat consumption with foodborne outbreaks in PNG but consumption of contaminated chicken meat has been identified as one of the most important food vehicles for these organisms worldwide [4]. Enteric pathogens such as *Salmonella* and *Campylobacter* species are the most commonly isolated enteropathogens from human gastroenteritis cases [5]. A recent report suggested that the NSP from an edible plant source was able to inhibit the invasion of *Salmonella Typhimurium* across the chicken intestine [6]. Also, in PNG, the most common and financially devastating disease in modern broiler flocks is necrotic enteritis (NE) which is caused by *Clostridium perfringens*. This organism is considered to be part of the normal gut microflora of poultry, and predisposing factors must be present to produce clinical NE [7]. Intestinal mucosal damage by coccidiosis in chickens is usually considered one of the most important predisposing factors in poultry, other than nutrition or diet.

Our hypothesis was that the inclusion of sweetpotato (SP) in broiler diets can influence the shedding of pathogens such as *Clostridium perfringens*, *Salmonella* and *Campylobacter* without affecting broiler performance. Also, there is little published information on the presence of enteropathogens in poultry flocks in PNG. This trial was conducted in PNG to study the effects of the inclusion of different sweetpotato varieties with varying levels of NSP content in a broiler diet on the general performance and levels of *Salmonella*, *Campylobacter* and *Clostridium* species (spp.). 

## 2. Materials and Methods

The current study was undertaken in Lae, Morobe Province, in PNG at the Feed Testing Facility of the National Agricultural Research Institute. The experiment was conducted between April and May of 2014. The average daily temperature for that period was 28 °C with a relative humidity of around 80%. Minimum and maximum temperatures recorded for that period were 26 °C and 30 °C, respectively. The study was approved by Animal Ethics Committee of the University of Adelaide, AEC Project No.S-2014-026. All procedures complied with the Australian Code of Practice for the Care and Use of Animals for Scientific Purposes. 

### 2.1. Sweetpotato Diets

Five commercial sweetpotato varieties were purchased from various provincial markets in the Highlands and Morobe Provinces of PNG. These varieties were identified by their local names; Waghi besta (Variety 1), Gimane (Variety 2), Suga (Variety 3), Marasonda (Variety 4) and Rachel (Variety 5). Briefly, fresh sweetpotato roots were washed, sliced and oven (Vision Scientific Company, Korea) dried at 60 °C for 16 h. The dried chips were milled, packed in bags and stored in storage bins until use. Representative samples (500 g) of these varieties were packed into labelled plastic bags and sent to the Institute of Subtropical Agriculture, The Chinese Academy of Sciences, P.R. of China for the analysis of total NSP and compositional sugars as described by Englyst and Cummings [8] and Englyst, et al. [9]. Moisture content, crude protein, fat, ash content, acid detergent, and neutral detergent fiber and total starch were analyzed by Agrifood Technology., Melbourne, Australia. The chemical methods used were based on the Australian Fodder Industry Association (AFIA) methods [10]. Gross energy values of the assay diets were determined using the PARR 6420 Automatic Isoperibol Calorimeter (John Morris Scientific, Adelaide, Australia). Sorghum, soybean, sunflower oil, limestone, di-calcium phosphate, salt, and a mineral and vitamin premix were sourced from PNG commercial suppliers in ground form. A sorghum-soybean meal basal diet was formulated, and five assay diets were developed to replace 250 g/kg of the basal diet with each of the five varieties of sweetpotato with and without enzyme inclusion. The specific enzyme product used in this study was Rovabio Excel AP T-flex (Endo-1,4-beta-xylanase, endo-1,3,9(4)-beta-glucanase) sourced from BEC Feed solutions (Brisbane, Australia). The enzyme product used was included as a powder at 50 g/1000 kg as per the manufacturer’s instructions. The composition of the basal diets is shown in Table 1 while the chemical composition of the five tested sweetpotato varieties is presented in Table 2. The total NSP content of the tested sweetpotato varieties ranged from 162–210 g/kg dry matter (DM, Table 2). Varieties with total NSP contents below 180 g/kg DM were considered to be low NSP varieties while varieties with total NSP contents above 200 g/kg DM were considered to be high NSP varieties. Varieties with total NSP content between 180–200 g/kg were considered to be varieties with medium levels of total NSPs.

### 2.2. Experimental Chicks, Diets, and Design

A total of 260 ROSS 308 broiler day-olds were bought from a local hatchery (Nuigini Table Birds) and reared on deep litter in two separate floor pens in an open, naturally ventilated shed from day-old to 20 days of age. The chicks were fed with a standard commercial broiler starter crumble (26.2% protein, 5.8% fat, 3.2% fiber and 7.1% ash content) up until the start of the experiment at day 21. Data loggers were placed inside the pens for automated recordings of daily room temperatures and relative humidity during the experimental period. Mortality and temperatures were also recorded at regular intervals (0600h, 0100h, 1200h, 1500h, and 1800h). At the start of the assay on day 21, the average weight of the flock was determined so that the individual weight of the experimental birds used in the trial was within ±200g of the average flock weight. After calculating the average body weight, 4 birds at a time were weighed and allocated to each of the 48 metabolic cages, measuring 600 × 450 × 380mm. The experimental design was a completely randomized design with sweetpotato and enzymes as the two main treatments factors. The experimental treatments were replicated four times. A total of 12 experimental treatments (including the control) were tested and these were Basal − (Control), Basal + Enzyme − (Control (+E)), Sweetpotato Variety 1 (SPV1), Sweetpotato Variety 1 + Enzyme (SPV1 (+E)), Sweetpotato Variety 2 (SPV2), Sweetpotato Variety 2 + Enzyme (SPV2 (+E)), Sweetpotato Variety 3 (SPV3), Sweetpotato Variety 3 + Enzyme (SPV3 (+E)), Sweetpotato Variety 4 (SPV4), Sweetpotato Variety 4 + Enzyme (SPV4 (+E), Sweetpotato Variety 5 (SPV5), Sweetpotato Variety 5 + Enzyme (SPV5 (+E). These experimental diets were fed to 4 experimental birds housed together in a group metabolic cage. All the cages had individual feed troughs and nipple drinkers and each cage was shielded to prevent the cross-contamination of excreta from the neighboring cages. The Apparent Metabolizable Energy (AME) bioassay was conducted as described by Hughes [11] using 21-day-old broiler chickens. Feed intake and excreta output were measured quantitatively per cage from day 25 for four consecutive days. Excreta collections from each cage were oven dried overnight daily and then pooled. Pooled dried excreta were ground and a representative sample taken for the determination of gross energy for the calculation of AME values of these diets. 

### 2.3. Tissue Sample Collection

At the end of the experiment, for each cage at a time, individual birds were weighed and one bird per cage based on the average group weight was selected for gut morphology and cecal bacterial enumeration. The selected birds were euthanized by cervical dislocation. Final body weights of the birds were recorded prior to dissection. Gut tissues and cecal contents were aseptically collected. A section of jejunum (before Meckel’s diverticulum) with contents were weighed and then flushed with phosphate buffer saline and 5 cm was collected for gut morphology studies. The tissue samples were fixed in 10% buffered formalin and later transferred to 70% ethanol for further processing. Two slides were prepared for each tissue. Cecal contents were collected into a sterile tube and immediately placed on ice for bacterial cultivation and enumeration.

### 2.4. Bacterial Isolation, Enumeration and Detection and Quantification by Quantitative Real-Time PCR 

For enumeration of *Escherichia coli (E.coli)* and total enterobacteria, one gram of cecal content from each euthanized bird was placed into a sterile tube containing 9 mL of sterile phosphate buffered saline and homogenized. Subsequent 10-fold serial dilutions for each sample were prepared to 10^−7^ dilutions. Then 0.1-mL of samples from 10^−3^, 10^−4^ and 10^−5^ dilutions were inoculated onto MacConkey and Violet Red Bile Glucose media (Oxoid, Australia) for enumeration of *Escherichia coli* and total enterobacteria, respectively. All plates were incubated at 37 °C for 24 h, after which total numbers of bacterial colonies were manually counted. The counts were converted into logarithmic equivalents and expressed as number of colony forming units CFU/g of cecal content. Due to lack of appropriate facilities and frequent power outages in-country (PNG), efforts to culture *Campylobacter* and Clostridium failed.

### 2.5. DNA Extraction from Caecal Contents

Extraction of DNA from cecal contents was performed using a QIAamp DNA fast DNA stool kit (Qiagen, Hilden, Germany) as per the manufacturer’s instruction. The eluted DNA concentration and quality was determined using a Nano drop (ThermoFisher Scientific, Massachusetts, MA, USA). The DNA was then diluted with PCR water to 5 µg/µL and stored at −20 °C until required. DNA extraction was performed in PNG. The DNA samples were imported into Australia (DAFF Import permit number IP14001999).

### 2.6. Quantitative Real-Time PCR Assay

The details of primers used in the quantitative real-time PCR reactions, is described in Table 3. To investigate the detection limit of *Salmonella*, *Campylobacter*, and *Clostridium* spp., for each bacterium at a time, 200 mg of fecal samples were spiked with each of the specific bacteria at doses ranging from 10^0^ to 10^8^ CFU/mL. DNA was then extracted from these spiked samples using QIAamp DNA Stool Mini Kit (Qiagen, Australia). The quantitative PCR reaction for the detection of the specific bacteria (Salmonella, *Campylobacter*, and Clostridium spp.) was performed in a total reaction volume of 10 µL containing 5 µL of 1× SYBR Green Master mix (Qiagen, Australia), 0.5 µL each of the forward and reverse primers of the specific target gene, 2 µL of PCR water and 2 µL of sample DNA diluted to 5 µL/µg. Each reaction was performed in duplicate and the reaction volume was dispensed using an automation workstation (Corbett Robotics, Australia). The Quantitative PCR (QPCR) assays were conducted in a Rotor-Gene 6000 Series real-time PCR machine (Corbett, Sydney, Australia) under the following conditions; 95 °C for 10 seconds, followed by 40 cycles of 95 °C for 10 s and 55 °C for 30 s. 

To determine the detection limit of the QPCR reaction for each specific bacterium, the reaction was run against a set of standards generated with the spiked samples of known concentrations. The detection limit of *C. perfringens* was 100 CFU while detection limit of *Campylobacter* and *Salmonella* was 1000 CFU.

### 2.7. Histopathology

Intestinal tissues were processed based on procedures described by Iji, et al. [15]. Slides were viewed on an Olympus BH-2 microscope at ×4 magnification and digitized using an image analysis software (AnalySIS 5 Soft-Imaging System, Hamburg, Germany). Each individual image was calibrated accordingly and measurements (micrometer) of villus height, crypt depth, basal villus width, and apical villus width were carried out on 10 intact villi. Apparent villus surface area was estimated from the trigonometric relationship between villus height, villus basal width and villus apical width [15].

### 2.8. Statistical Analysis

Analysis of the data was performed using the two way analysis of variance in GenStat^®^, 15th Edition [16]. The difference between the means was identified by the least significant difference and compared using the Duncan’s multiple range test. 

## 3. Results

### 3.1. Total Enterobacteria Counts and Real-Time PCR Counts of Campylobacter, Clostridium, and Salmonella Species

There were no significant differences (*p* < 0.05) in the colony counts of total enterobacteria and *E. coli* in the ceca of broilers due to the different dietary treatments (Table 4). The counts of total enterobacteria ranged from 2.301 to 5.602 log CFU/g while the counts of *E.coli*, ranged from 4.998 to 5.936 log CFU/g of cecal contents. Birds fed with the SPV5 diet had the lowest count of total enterobacteria while the highest count was observed in birds fed with the SPV1 (+E) diet. The lowest count for *E.coli* was observed in birds fed with the SPV3 diet (4.998 log CFU/g) while the highest count was observed in birds fed with the SPV1 diet. There were no significant interactions between the different SP varieties and enzyme inclusion on the numbers of these bacterial species in the ceca of birds fed the different diets (Table 4).

Results from the real-time PCR assays on the levels of *Campylobacter, C. perfringens and Salmonella* are presented in Table 5. The load of *Campylobacter* (log CFU/g) in the ceca of birds fed the different dietary treatments was significantly (*p* = 0.004) influenced by the SP varieties. Inclusion of enzymes did not significantly (*p* = 0.181) influence the load of this enteric pathogen in the ceca of birds. There was, however, a significant interaction (*p* = 0.020) between the different SP varieties and enzyme supplementation on the loads of *Campylobacter* (log CFU/g). The load of *Campylobacter* (log CFU/g) in birds fed with the SPV3, SPV4, and SPV5 diets was reduced with enzyme supplementation (Table 5). The lowest level of *Campylobacter* was observed in birds fed with the SPV3 diet (1.271 log CFU/g) while the highest was observed in birds fed with the SPV5 diet (5.831 log CFU/g). The levels of *Campylobacter* in birds fed with the SPV5 diets were reduced by 76.4% with enzyme supplementation.

As shown in Table 5, the loads of *C. perfringens* in the ceca of birds were significantly (*p* ≤ 0.001) influenced by the SP varieties but not by enzyme supplementation (*p* = 0.903). There was no interaction between the enzyme and the different SP varieties. Birds fed with the SPV3 diet had a significantly lower *C. perfringens* load compared to those observed in the ceca of birds fed with the other treatment diets. The load of this enteric pathogen ranged from 2.050 to 3.526 log CFU/g of cecal content. The highest level of this pathogen was observed in the ceca of birds fed with the SPV2 diet. The load of *C. perfringens* in birds fed with this diet (SPV2) was reduced by 3.86% with enzyme supplementation. The levels of this pathogen were however elevated with enzyme inclusion.

The numbers of *Salmonella* spp. were not significantly influenced by the SP varieties (*p* = 0.758) or with the enzyme supplementation (*p* = 0.685) (Table 5). However, there was a significant (*p* = 0.047) interaction between the enzyme supplementation and the SP varieties. The load of *Salmonella* ranged from 0.00 to 1.753 log CFU/g of cecal content. The lowest level was observed in birds fed with the control diet while the highest was observed in the ceca of birds fed with the SPV5 diet. Inclusion of the enzyme reduced the load of *Salmonella* in the ceca of birds fed with the different SP varieties. Inclusion of the enzyme in the control diet significantly elevated the levels of *Salmonella* while the inclusion of enzymes in the SPV5 diet reduced the load of this pathogen by 57.2%. 

### 3.2. NSP Levels of the Different Sweetpotato Varieties, AME Values of the Diets and Growth Performance of Broilers

The total NSP content of the tested sweetpotato varieties from PNG ranged between 162 and 210 g/kg DM (Table 2). Varieties 3 and 5 had the highest total NSP content of 202 and 210 g/kg DM respectively while variety 2 had the lowest total NSP content of 162 g/kg DM. Varieties 1 and 4 had the total NSP content of 197 and 190 g/kg DM, respectively. The differences in the proportions of the soluble and insoluble fractions of the total NSP in the tested varieties is evident both within and between each variety (Table 2). 

There were significant differences due to the SP varieties and enzyme supplementation (*p* < 0.001) on the AME values of the different SP diets (Table 6). A significant interaction (*p* < 0.001) between the sweetpotato varieties and enzyme supplementation was also observed (Table 6).

The SPV2 diet had the lowest AME value while the highest AME was recorded for the Control (+E) diet. In terms of the SP varieties, SPV1 diet had a higher AME value (15.46 MJ/kg) compared to other SP diets. There was an improvement in the AME of variety 2 with the enzyme inclusion (11.22 to 13.97 MJ/kg). 

### 3.3. Growth Performance of Birds Fed Different SP Diets

In terms of bird performance, significant differences were observed on the end weight (*p* = 0.06), weight gain (*p* < 0.001), feed intake (*p* < 0.001) and feed conversion ratio (FCR) (*p* = 0.001) of birds fed the different diets (Table 7). There were significant interactions between the SP varieties and enzyme inclusion on the intake and FCR of birds. Intake of birds fed with the SPV3 diet was improved with enzyme inclusion. Birds fed with the Control (+E) and the SPV1 (+E) diets had significantly higher intakes compared to birds fed with the other diets. In terms of FCRs, birds fed with the SPV1 (+E) diet had significantly higher FCR which is indicative of a high intake and lower weight gains. FCRs of birds fed with the other four SP diets were comparable to that of the birds fed with the Control (+E) diet. As expected, birds fed on the control diet had a significantly lower FCR compared to that of the birds fed on the other test diets (Table 7).

### 3.4. Relative Organs Weights and Jejunal Morphology

There were no significant differences (*p* > 0.05) observed on the relative weights of the liver, pancreas and segments of the small intestine of broilers fed with the different SP diets (Table 8). Inclusion of the enzymes in the diets did not significantly influence the above-mentioned parameters. There was no interaction between the enzymes and the SP varieties on the relative organ weight. 

In terms of the jejunal morphology, there were no significant differences (*p* > 0.05) observed in the villus height, villus crypt depth, villus height and crypt depth ratio and the apparent surface area in the birds fed with the different sweetpotato varieties (Table 9). The mean villi heights of the jejunum from the birds fed with the different diets ranged from 1105 to 2132 µm, while the mean crypt depth measurements ranged from 121 to 246 µm. The highest apparent surface area (0.402 mm^2^) was observed in birds fed with the SPV3 (+E) diet. There were no significant (*p* > 0.05) differences observed with enzyme inclusion on the measured parameters (Table 9).

## 4. Discussion

The aim of this study was to investigate the shedding of *Campylobacter*, *C. perfringens* and *Salmonella* spp. in birds fed with the different SP varieties with differing NSP contents. Growth performance of the birds and the gut morphology of selected birds were also assessed. 

Different dietary interventions may affect the health of birds as the microbial status of the gastrointestinal tract of poultry is influenced by the diet composition and the internal gut environment [7,17]. The commensal microbial community of the gut plays a major role in the health and digestion of poultry [18], therefore any dietary interventions can either increase or decrease the risk of infection by enteric pathogens. 

### 4.1. Effects of SP Varieties on Salmonella spp., Campylobacter, and C. perfringens

In the current study, the counts (log CFU/g) of total enterobacteria and *E.coli* from the ceca of birds fed with the different dietary treatments were not significantly influenced by the different SP varieties and enzyme supplementation. This observation is similar to our earlier experiment [19] using sweetpotato flour in which the counts of the total enterobacteria and *E. coli* were not influenced by the inclusion of sweetpotato flour or enzyme supplementation. 

In the present study, the load (log CFU/g cecal content) of *Campylobacter* was significantly influenced by the different SP varieties but not by the inclusion of enzymes. There is, however, a significant interaction between the different SP varieties and enzyme supplementation on the loads of *Campylobacter* (log CFU/g). The load of this pathogen in the ceca of birds was reduced when the enzymes were included in the SPV3, SPV4, and SPV5 diets. The mentioned varieties (Varieties 3, 4 and 5) had medium to high levels of total NSP content (Table 2). Earlier studies reported that there was a reduction in the numbers of *Campylobacter* in the ceca of broilers fed with a wheat-based diet and NSP degrading enzymes [20,21]. 

In the current study, the levels of *Salmonella* spp. were not significantly influenced by the SP varieties or with enzyme supplementation but a significant interaction between the enzyme supplementation and the SP varieties was observed (Table 5). Teirlynck, et al. [22], suggested that a possible way to control *Salmonella* in broiler chickens was to change the carbohydrate composition, specifically the NSP content in diets for broilers. In addition to that, the supplementation with an enzyme product can decrease the level of *Salmonella* in broilers fed these diets as the hydrolysis of the NSPs by these enzymes will give lead to the production of short chain fatty acids will (SCFA) [23]. These SCFAs are known to have bacteriostatic effects [23,24].

The levels of *Campylobacter* and *Salmonella* were highest in the birds fed with the SPV5 diet without the enzyme supplementation. This variety had the highest total NSP content (210 g/kg) with only a marginal difference in the amount of soluble and insoluble NSP components (106 g/kg vs. 104 g/kg DM). Similar to *Campylobacter*, the levels of *C. perfringens* in the ceca of birds were significantly influenced by the SP varieties but not with enzyme supplementation. *C. perfringens* levels were high in the ceca of birds fed with the SPV2 diet without the enzyme supplementation. This variety had a low total NSP content (162 g/kg DM) (Table 2). This contrasts with the levels of *Campylobacter* and *Salmonella* which were highest in the birds fed with the SPV5 diet without the enzyme supplementation. This SP variety has a higher total NSP content (210 g/kg DM). There is limited data available on the levels and the shedding of these enteropathogens in broilers fed with sweetpotato diets in PNG. The only available data to date is a study by Tasi (2015) which showed that *Campylobacter* and *Salmonella* are present in poultry farms in PNG, but levels were significantly lower compared to those reported in developed countries. The results of that study showed that *Campylobacter* and *Salmonella* were detected in 36.3% and 1.5% of the chicken meat samples, respectively. The levels of the *Campylobacter* and *Salmonella* in the present study is similar in that the levels of *Salmonella* were lower compared to that of the *Campylobacter* in the ceca of broilers fed on the SP diets in PNG.

Soluble NSPs in grains such as wheat, barley and rye are known to create a viscous gut environment in broilers leading to a reduced digesta transit time which creates a conducive environment for the proliferation of pathogens [25,26,27,28,29]. In addition to that, enzyme supplementation is associated with the production of SCFAs due to the fermentation of NSPs in the ceca of birds [30,31]. Production of these SCFA would have made the environment more acidic thus reducing the numbers of *Campylobacter and Salmonella* while *C. perfringens* levels may not have been affected as species belonging to the Clostridiaceae family in the lower intestinal tract are butyrate producers [22].

The regular use of alternative feed ingredients in the diets for poultry is impeded by high fiber fractions as these fiber fractions are structural carbohydrates that are not digested by the endogenous enzymes in poultry and other monogastric livestock [32]. The use of exogenous enzymes aids the hydrolysis of this feed component, thus reducing the gut viscosity in poultry [33]. In the current study, inclusion of the enzymes to the SP varieties with a high total NSP content reduced the level of the *Campylobacter* and *Salmonella* species in the ceca of birds fed on those diets. Apart from the study by Parsons, Wigley, Simpson, Williams, Humphrey, Salisbury, Watson, Fry, O’Brien, Roberts, O’Kennedy, Keita, Söderholm, Rhodes and Campbell [6], there is limited information available on how NSPs in crops other than grains, influence the gut microbiota, which may then affect growth performance of broilers raised under less than perfect management conditions. *Campylobacter* spp., *C. perfringens* and *Salmonella* spp. are important zoonotic bacteria that infect humans and these pathogens are part of the normal gut microbiota in chickens [5]. The type of diet offered to broiler chickens is vital to reducing the bacterial contamination in the end product (chicken meat in this case). In the current study, the numbers of *Campylobacter* and *Salmonella* spp. were higher in the ceca of birds fed with the SP varieties that had high NSP content while *C. perfringens* was highest in birds fed with the SP variety with a low total NSP content. There is an obvious link to the proportion of soluble and insoluble fiber components influencing the load of these enteropathogens in the ceca of broilers. In the present study, the numbers of *C. perfringens* in the sweetpotato diets were comparable to that of the birds fed on the control diet and were not influenced by the inclusion of the enzyme. There could be three underlying possibilities for this finding; 1) the NSPs of these sweetpotato varieties are not viscous, 2) the levels of insoluble NSPs are higher than the soluble component or 3) the diets are highly digestible. 

In the present study, the AME values of the different SP diets ranged from 12.38 to 15.46 MJ/kg and can be attributed to the total NSP content of these varieties and place of origin (PNG). The dry matter digestibility (DMD) of the sweetpotato diets showed that these dietary treatments were well used by the birds (Table 6) and may be due to the diets being offered as pellets [34,35] as pelleting sweetpotato diets is necessary due to its dusty texture [36].

### 4.2. AME Values for SP Varieties and Effects on Production Parameters 

The relationship between AME in wheat diets and the FCR of broilers is often negatively correlated with soluble NSP and soluble arabinoxylan content [25,28,37]. There is limited information available on the NSPs of roots and tuber crops, and how they may impact on the overall AME of the diet and the FCR of broilers. In the current study, we observed a lower AME value in the SPV2 (Gimane) diet. This variety had a low total NSP content but a higher soluble NSP component (Table 2) and this is similar to that observed in wheat, where varieties with high soluble NSP content had lower AME values [11,38]. The other four SP diets had a medium to high total NSP content however their soluble NSP content were lower (Table 2). The AME value of SP 2 was greatly improved with enzyme inclusion. The use of exogenous enzymes aids the hydrolysis of soluble NSP leading to reduced gut viscosity in poultry, which in turn enables other nutrients to be readily available for absorption [39]. Currently, the majority of the commercial exogenous enzyme products are targeted at the soluble component of feed ingredients. Insoluble NSPs particularly cellulose are not a practical target for improvement in poultry as no enzyme can effectively release glucose from this component, nor does insoluble NSP create a viscous gut environment in birds. This may be the case here as AME values of varieties with higher proportions of insoluble NSP were negligible (Table 6). 

The inclusion of sweetpotato in the present study had a significant influence on the production parameters such as the body weight gain, feed intake and the FCR of broilers. Of the sweetpotato varieties tested, birds fed with the SPV3 diet had an FCR that was comparable to that of the birds fed on the control diet. This variety had a higher insoluble NSP component and may have enhanced gut digestive parameters [40]. 

Gut morphology in the jejunum of the birds fed with this variety had a higher villi height and a larger apparent surface area available for absorption. The inclusion of sweetpotato did not have any significant effect on the gut morphology of birds in the current study (Table 9). The gut morphology (villi height, crypt depth and the apparent surface area) in the jejunum of the birds with a high load of *Campylobacter* and *Salmonella* (birds fed with the SPV5 diet) and *C. perfringens* (birds fed with the SPV2 diet) were not affected. 

## 5. Conclusions

In conclusion, given that sweetpotato will be used extensively in diets for poultry in PNG and other Pacific Island nations in the near future, this study provides useful insights on the effects of feeding local SP varieties on level of food borne pathogens in chickens. Birds fed the sweetpotato diets without the inclusion of enzymes had higher bacterial cell counts for all three enteropathogens. The level of *C. perfringens* was higher in the birds fed with the sweetpotato variety with a low total NSP content. Given that the numbers of *C. perfringens* were lower in the gut of broilers fed with these varieties their inclusion in the feed is likely to reduce the risk of NE. On the other hand, *Campylobacter* and *Salmonella* levels in the broilers fed with the sweetpotato diets can be reduced with enzyme inclusion. To our knowlegde this is the first study conducted in PNG to assess the levels of enteric pathogens such as *Salmonella*, *Campylobacter*, and *Clostridium* spp., in the smallholder poultry sector of PNG. The NSP levels in sweetpotato did not affect the gut morphology of birds which highlights the possibility of using different sweetpotato varieties for feeding poultry. In terms of the total NSP content, selection of varieties with a low soluble NSP content would be economical as AME values are within the range selected for poultry without the need for enzyme inclusion. This information will be useful for policy makers in addressing food safety issues in the country. In addition to that, the information could also help for inclusion and or selection of local feed ingredients for reducing the load of enteric pathogens. 

## Figures and Tables

**Table 1:** Composition of the basal diet (g/kg).

Ingredient	Basal
Sorghum	57.50
Soybean meal	32.00
Sunflower oil	6.00
Di-calcium phosphate	2.00
Limestone	1.10
DL-methionine	0.70
Vitamin and mineral premix	0.20
Salt	0.30
Choline chloride (60%)	0.20

**Table 2:** Chemical composition of 5 local Papua New Guinea (PNG) Sweetpotato cultivars.

Measured Nutrient Content	Variety 1	Variety 2	Variety 3	Variety 4	Variety 5
Dry Matter (% DM)	90.2	90.1	89.1	91.3	83.5
Protein (N × 6.25) (% DM)	4.1	3.8	4.8	3.4	2.4
Fat (DM)	2.5	1.9	1.7	1.8	0.68
Ash (DM)	2.7	2.6	2.1	2.3	1.8
Total Starch (% DM)	59	53	58	62.4	-
Non-starch polysaccharides (g/kg)					
Soluble	69	94	64	81	106
Insoluble	128	68	138	109	104
Total	197	162	202	190	210

Variety 1 = Waghi besta; Variety 2 = Gimane; Variety 3 = Suga; Variety 4 = Marasonda; Variety 5 = Rachel.

**Table 3:** Description of primers selected for Q-PCR reactions for the detection of *Campylobacter*, Clostridium, and Salmonella species.

Bacteria	Target Gene	Primer	Gene Sequence	Size (Bp)	Source
*Campylobacter*	16S rRNA	F	CATTGGATGAGCAACTTAAAATC	125	[12]
		R	TTCTTCCATTAAACGGTTGA		
*Clostridium perfringens*	16SrRNA	CPerf165F	CGCATAACGTTGAAAGATGG	105	[13]
		CPerf269R	CCTTGGTAGGCCGTTACCC		
*Salmonella* spp.	*invA* gene	F	CATTTCTATGTTCGTCATTCCATTACC	132	[14]
		R	AGGAAACGTTGAAAAACTGAGGATTCT		

**Table 4:** Counts of total Enterobacteriaceae and E.coli (Log CFU/g/mL) in chickens fed different sweetpotato diets.

Treatment	Enzyme	Enterobacteria	*Escherichia coli*
SP Variety			
Control	(−)	5.079	5.547
	(+)	5.590	5.694
SPV1	(−)	5.201	5.936
	(+)	5.602	5.865
SPV2	(−)	2.540	5.402
	(+)	5.345	5.132
SPV3	(−)	5.429	4.998
	(+)	4.801	5.457
SPV4	(−)	5.318	5.312
	(+)	2.628	5.304
SPV5	(−)	2.301	5.380
	(+)	5.102	5.529
SEM		1.263	0.369
Source of variation			
SP variety		*0.588*	*0.467*
Enzyme		0.479	0.756
SP variety × Enzyme		*0.298*	*0.945*

Means are not significantly different (*p* < 0.05); SEM = Standard error of means.

**Table 5:** Log CFU (g/mL) of cecal content of chickens fed different sweetpotato diets.

Treatment	Enzyme	*Campylobacter*	*C. perfringens*	*Salmonella* spp.
SP Variety				
Control	(−)	4.095 ^abc^	3.421 ^a^	0.000 ^b^
	(+)	5.734 ^a^	3.514 ^a^	1.635 ^a^
SPV1	(−)	4.840 ^ab^	3.181 ^a^	0.970 ^ab^
	(+)	3.733 ^abc^	2.940 ^a^	0.995 ^ab^
SPV2	(−)	2.461 ^bc^	3.526 ^a^	1.003 ^ab^
	(+)	2.882 ^abc^	3.390 ^a^	0.852 ^ab^
SPV3	(−)	1.271 ^c^	2.050 ^b^	0.680 ^ab^
	(+)	2.077 ^bc^	2.788 ^a^	0.440 ^ab^
SPV4	(−)	2.980 ^abc^	3.138 ^a^	1.493 ^ab^
	(+)	1.375 ^c^	2.905 ^a^	1.093 ^ab^
SPV5	(−)	5.831 ^a^	3.292 ^a^	1.753 ^a^
	(+)	1.376 ^c^	2.977 ^a^	0.224 ^ab^
SEM		0.919	0.222	0.469
Source of variation				
SP variety		0.004	<0.001	0.758
Enzyme		0.181	0.903	0.685
SP variety × Enzyme		0.020	0.173	0.047

Means within a column with different superscript are significantly different (*p* < 0.05); SEM = Standard error of mean.

**Table 6:** AME values and dry matter digestibility of treatment diets.

Treatment	Enzyme	AME DM (MJ/kg)	DMD
SP Variety			
Control	(−)	13.97 ^b^	0.656 ^b^
	(+)	15.99 ^a^	0.785 ^a^
SPV1	(−)	15.65 ^a^	0.790 ^a^
	(+)	15.27 ^a^	0.778 ^a^
SPV2	(−)	11.22 ^c^	0.660 ^b^
	(+)	13.55 ^b^	0.662 ^b^
SPV3	(−)	13.39 ^b^	0.664 ^b^
	(+)	13.97 ^b^	0.683 ^b^
SPV4	(−)	13.802 ^b^	0.685 ^b^
	(+)	13.98 ^b^	0.690 ^b^
SPV5	(−)	13.634 ^b^	0.668 ^b^
	(+)	13.35 ^b^	0.666 ^b^
SEM		0.194	0.029
Source of variation			
SP variety		<0.001	<0.001
Enzyme		<0.001	0.008
SP variety x Enzyme		<0.001	<0.001

Means within a column with different superscripts are significantly different (*p* < 0.001; *p* < 0.05); DMD = Dry matter digestibility; SEM = Standard error of means.

**Table 7:** Summary of main effects of diet and enzyme on performance parameters of broilers.

Treatment	Enzyme	Start Weight (g)	End Weight (g)	Weight Gain (g)	Feed Intake (g)	FCR (g/g)
SP Variety						
Control	(−)	941.9 ^a^	1424 ^ab^	482.5 ^a^	946.0 ^b^	1.96 ^c^
	(+)	930.0 ^a^	1416 ^c^	486.2 ^a^	1326 ^a^	2.75 ^b^
SPV1	(−)	935.0 ^a^	1244 ^bc^	309.4 ^c^	1262 ^a^	4.09 ^a^
	(+)	945.0 ^a^	1233 ^c^	287.5 ^c^	1192 ^a^	4.25 ^a^
SPV2	(−)	948.8 ^a^	1267 ^bc^	318.1 ^bc^	919.1 ^b^	2.88 ^b^
	(+)	938.1 ^a^	1258 ^bc^	320.0 ^bc^	910.8 ^b^	2.88 ^b^
SPV3	(−)	955.6 ^a^	1261 ^c^	305.6 ^c^	869.0 ^b^	2.88 ^b^
	(+)	935.6 ^a^	1327 ^abc^	391.2 ^b^	986.9 ^b^	2.52 ^b^
SPV4	(−)	936.9 ^a^	1260 ^bc^	323.1 ^bc^	906.0 ^b^	2.83 ^b^
	(+)	945.6 ^a^	1246 ^c^	300.6 ^c^	907.9 ^b^	3.01 ^b^
SPV5	(−)	930.0 ^a^	1236 ^c^	306.2 ^c^	896.4 ^b^	2.93 ^b^
	(+)	941.9 ^a^	1269 ^c^	327.5 ^bc^	893.6 ^b^	2.79 ^b^
SEM		62.5	96.79	46.2	113.2	0.345
Source of variation						
SP variety		0.999	0.006	<0.001	<0.001	<0.001
Enzyme		0.913	0.739	0.400	0.04	0.298
SP variety × Enzyme		0.992	0.948	0.215	0.004	0.045

Means with the same letter within a main effect are not significantly different (*p* > 0.05); FCR = Feed conversion ratio; SEM = Standard error of means.

**Table 8:** Dietary effects on the relative organ and intestinal segments (g/kg) of broilers.

Treatment	Enzyme	Pancreas	Liver	Small Intestine	Duodenum	Jejunum	Ileum
SP Variety							
Control	(−)	2.52	23.54	49.32	12.17	22.06	15.1
	(+)	2.33	22.57	50.34	12.1	23.73	14.51
SPV1	(−)	2.67	24.65	52.45	12.76	23.39	16.3
	(+)	2.9	24.6	49.94	12.62	21.72	15.61
SPV2	(−)	3.07	28.22	50.93	11.69	23.51	15.72
	(+)	3.18	23.83	51.93	12.78	23.16	15.99
SPV3	(−)	3.06	23.67	51.32	12	23.89	15.44
	(+)	2.51	24.15	47.75	11.13	21.45	15.17
SPV4	(−)	3.29	26.7	50.3	11.12	23.23	15.94
	(+)	3.1	25.28	54.2	12.68	26.15	15.37
SPV5	(−)	3.11	24.33	51.2	12.35	22.81	16.04
	(+)	3.19	27.01	54.65	13.31	25.37	15.97
SEM		0.564	3.32	3.896	1.796	3.18	2.559
Source of variation							
SP variety		0.07	0.383	0.843	0.729	0.716	0.929
Enzyme		0.61	0.528	0.75	0.422	0.63	0.666
SP variety × Enzyme		0.776	0.436	0.749	0.753	0.427	0.999

Means are not significantly different (*p* < 0.05); SEM = Standard error of means.

**Table 9:** Dietary effects on morphological changes in the jejunum of broilers.

Treatment	Enzyme	Villus Height (µm)	Crypt Depth (µm)	Villus: Crypt Ratio	Apparent Surface Area (mm^2^)
SP Variety					
Control	(−)	1603	173.0	5.97	0.328
	(+)	1295	185.2	7.37	0.155
SPV1	(−)	1105	118.5	10.85	0.099
	(+)	1703	180.5	9.01	0.288
SPV2	(−)	1517	167.9	10.77	0.207
	(+)	1898	189.1	9.87	0.319
SPV3	(−)	1596	179.8	5.95	0.305
	(+)	2132	245.7	10.23	0.402
SPV4	(−)	1152	121.7	7.47	0.176
	(+)	1625	145.1	12.56	0.284
SPV5	(−)	1319	141.0	9.49	0.131
	(+)	1234	124.5	12.70	0.145
SEM		527.8	61.42	2.759	0.128
Source of variation					
SP variety		0.871	0.756	0.621	0.675
Enzyme		0.390	0.435	0.252	0.439
SP variety × Enzyme		0.936	0.984	0.758	0.784

Means are not significantly different (*p* > 0.05); SEM-Standard error of means.
